# Crystallization of Human Aquaglyceroporins for Neutron Diffraction Studies

**DOI:** 10.1021/acsomega.4c08284

**Published:** 2025-02-20

**Authors:** Sofia W. de Maré, Jonas Hyld Steffen, Julie W. Missel, Amalie Gerdt Laursen, Kamil Gotfryd, Zoë Fisher, Mahmood Reza Amiry-Moghaddam, Pontus Gourdon, Karin Lindkvist-Petersson

**Affiliations:** † Experimental Medical Science, Medical Structural Biology, BMC C13, Lund University, 221 84 Lund, Sweden; ‡ Department of Biomedical Sciences, University of Copenhagen, Blegdamsvej 3B, 2200 Copenhagen, Denmark; § Lund Protein Production Platform (LP3) and Department of Biology, Lund University, 223 62 Lund, Sweden; # Scientific Activities Division, European Spallation Source ERIC(ESS), 221 00 Lund, Sweden; ∥ Laboratory of Molecular Neuroscience, Division of Anatomy, Department of Molecular Medicine, Institute of Basic Medical Sciences, University of Oslo, Post Box 1105, Blindern, 0317 Oslo, Norway; ⊥ LINXS - Institute of Advanced Neutron and X-ray Science, 223 63 Lund, Sweden

## Abstract

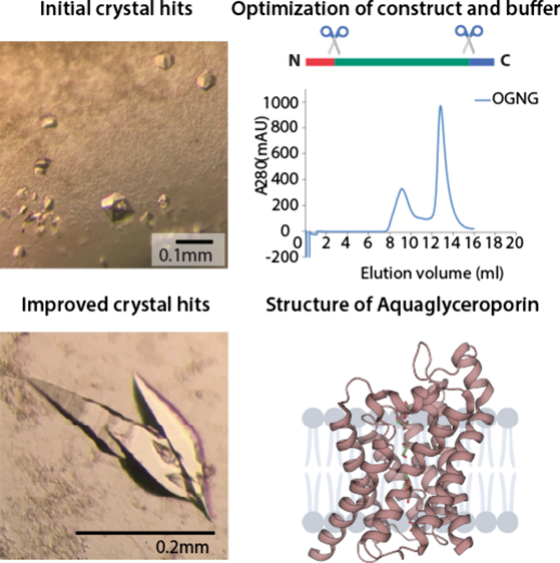

Aquaglyceroporins are channels that facilitate the flux of glycerol and water across lipid bilayers. Although structural information is available for several aquaglyceroporins, the details of how water and glycerol selectivity are maintained and how protons are excluded remain elusive. An approach to obtaining data on the hydrogen atom positions is to apply neutron macromolecular crystallography. Here, we present strategies to obtain large crystals suitable for neutron diffraction experiments by assessing a range of different methods, including new procedures for protein purification and crystallization. By applying long incubation times, macroseeding, and/or optimization of detergents, millimeter-sized crystals with different morphologies were obtained, and their diffraction quality was assessed by exposure to X-rays. The data presented here lay the foundation for continued crystallization efforts targeting aquaporins and other membrane proteins for neutron diffraction experiments.

## Introduction

Hydrogen atoms are fundamental in biology, as many processes critically depend not only on the overall fold of proteins but also on factors such as protonation states and hydrogen bond interactions.^1^ Detailed studies and understanding of protein function related to such matters require the knowledge of precise hydrogen atom positions. This information is, however, challenging to accurately obtain using conventional X-ray crystallography methods or cryo-EM. Neutron macromolecular crystallography (NMX) is a powerful complement to these techniques, with the additional benefit that the scattering of neutrons by hydrogen is comparable to that of other atoms in a protein, thereby facilitating the localization of hydrogen atom positions.^2^ Nevertheless, protein crystal structures determined by neutron diffraction remain severely under-represented in the protein data bank (PDB). To date, there are less than 250 structures determined using NMX, compared to more than 215,000 structures available in the protein data bank determined by other techniques. Furthermore, to our knowledge, only a single membrane protein, a bacterial ion channel, has been approached using neutron diffraction, yielding merely low-resolution data and no structure.^3^ The lack of membrane protein structures studied by NMX reflects the difficulties of obtaining large and well-diffracting crystals of membrane proteins, which are required due to the relatively low flux of the neutron beam.

**Figure 1:**
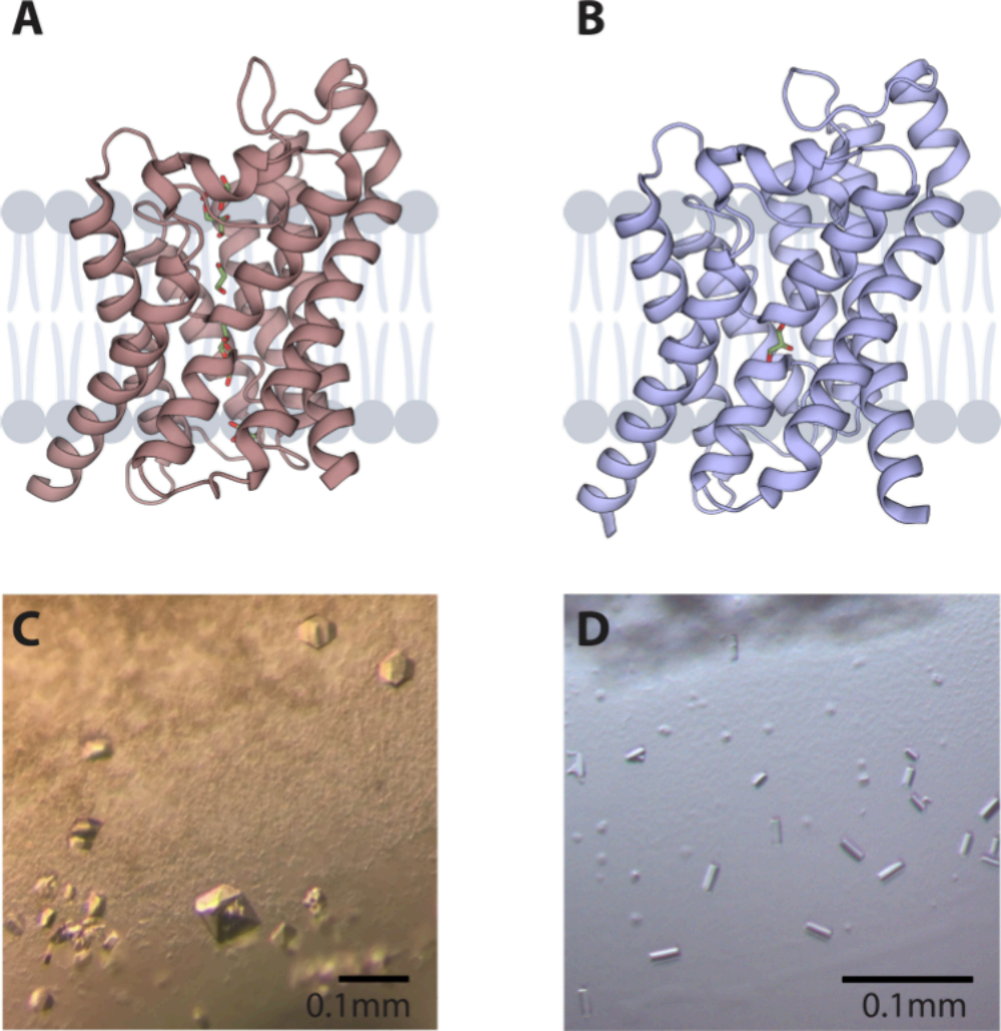
Structures and crystals of human AQP7 and AQP10. (A) AQP7 and (B) AQP10 are shown as cartoons, with glycerol molecules shown as sticks. (C) Crystals of AQP7 in OGNG with OG as an additive grown by hanging drop vapor diffusion crystallization. (D) Crystals of AQP10-ΔNΔC in NG grown by hanging drop vapor diffusion crystallization and NTG as an additive.

Aquaporins (AQPs) are integral membrane proteins that belong to one of the structurally best-characterized families of membrane proteins.^4^ The aquaporin family is commonly divided into three subgroups: the orthodox AQPs that allow passive water transport;^5^ the aquaglyceroporins that are permeable to solutes, such as glycerol, arsenic trioxide, hydrogen peroxide, and urea;^6^ and the less-studied superaquaporins with broader postulated solute specificity.^7^ There are 13 human aquaporins abbreviated as AQP0–12. We have previously determined the X-ray structures of human aquaglyceroporins AQP7 and AQP10, with water and glycerol molecules lining the pores (Figure 1A,B). Although AQP7 and AQP10 were determined at high resolution,^8,9^ the detailed selectivity mechanisms of aquaporins remain elusive due to the difficulties in precisely locating the hydrogen atoms, which is critical to understand the so-called proton exclusion mechanism. An analysis of the crystal structures of membrane proteins deposited in the PDB identified several proteins as potentially successful targets for NMX based on crystal size, unit cell parameters, and resolution.^10^ The crystals obtained for AQP7 and AQP10 meet these criteria with regard to resolution and unit cell dimensions, proposing that AQP7 and AQP10 are suitable targets for NMX studies. However, NMX requires substantially larger crystals compared to X-ray crystallography due to the inherently lower flux at neutron source instruments (<10^8^ neutron per mm^2^ per second) compared to standard photon flux at synchrotrons (>10^12^ per 100 μm^2^ per second),^11^ and the diffraction intensity depends on the incident beam in a directly proportional manner and inversely proportional to the unit cell volume squared.^2^ Here, with the aim to eventually study these targets using neutron diffraction, we present approaches and strategies for how to obtain crystals suitable for neutron diffraction studies of human aquaglyceroporins that are likely to be applicable to other membrane proteins.

## Experimental Procedure

### Protein Expression and Purification

Human AQP7 variants (wild-type, C-terminally, and C- and N-terminally truncated) were expressed in the methylotrophic yeast *Komagataella pastoris* (previously known as *Pichia pastoris*) and purified following a well-established protocol.^12^ In short, the cells were grown in flasks in a shaking incubator, the protein expression was induced by methanol for 30 h, and the cells were lysed using a high-pressure device at −20 °C. Membranes were solubilized in 1% n-decyl-beta-maltoside (DM) or 1% n-dodecyl-β-d-maltoside (DDM) for 1 or 2 h, respectively, at 4 °C, and the protein was purified by batch-wise affinity chromatography based on the C-terminal 6-His-tag, followed by size-exclusion chromatography (SEC) using a Superdex 200 Increase column (Cytiva). Detergents were present during the entire purification process at concentrations of at least 1.2 times the critical micelle concentration (DM: 0.10%, DDM: 0.03%, n-undecyl-β-maltoside (UDM): 0.03%, n-octyl β-d-maltoside (OM): 1.00%, decyl maltose neopentyl glycol (DMNG): 0.03%, n-octyl β-d-glucopyranoside (OG): 0.64%, n-nonyl β-d-glucopyranoside (NG): 0.24%, octyl glucose neopentyl glycol (OGNG): 0.07%, octyl β-d-1-thioglucopyranoside (OTG): 0.03%). The detergent was exchanged from DM in the detergent screening at the SEC column. Protein was concentrated using centrifugal filters with a 100 kDa cutoff (Amicon).

Human AQP10 variants (wild-type and N- and C-terminally truncated) were produced in *S. cerevisiae*, as previously described.^13^ Cells were grown in 10 L bioreactors, controlled by pH (6.0 using NaOH). The culture was inoculated with 2% glucose, followed by the addition of 3% glucose after approximately 24 h. Upon stagnation, calculated by the lowered addition of NaOH, the temperature was lowered to 15 °C and induced for 48 h with 2% galactose. AQP10 was isolated using a previously published protocol.^9^ Upon cell lysis using bead beating, the isolated crude membranes were solubilized using 2% DM for 4 h at 4 °C. AQP10 was purified by IMAC utilizing the C-terminally fused 10-His-tag in 0.2% DM, followed by SEC using a Superdex 200 Increase column (Cytiva). Upon SEC, DM was exchanged to detergents relevant for crystallization (NG: 0.4%, OG: 1%, n-nonyl-β-d-thioglucoside (NTG): 0.8%, OTG: 2%). AQP10 was concentrated using centrifugal spin columns with a 100 kDa cutoff (VivaSpin, Satorius).

### Crystallization

AQP7 was crystallized by hanging drop and sitting drop vapor diffusion in 24-well plates sealed with vacuum grease at 4, 18, and 30 °C. The protein concentration ranged between 2.5 and 14 mg·mL^–1^ in various detergents, as identified in the detergent screening. Initial crystallization was performed using commercial screening solutions (Molecular Dimensions, Hampton Research). Crystallization conditions were optimized by the systematic grid, additive, and detergent screening (Hampton Research) and by varying the protein concentration and protein/precipitant ratio in the drop. Crystals were cryoprotected by soaking in 20% glycerol in cases when the precipitant solution did not contain any cryoprotecting components.

AQP10 was crystallized by hanging drop and sitting drop vapor diffusion in 24-well plates sealed with immersion oil at 4 and 18 °C. The protein concentration ranged between 3 and 10 mg·mL^–1^. Initial crystallization was performed using commercial screening solutions (Molecular Dimensions, Hampton Research). Crystallization conditions were optimized by the systematic grid, additive, and detergent screening (Hampton Research) and by varying the protein concentration.

### X-ray Diffraction Testing

Crystals were tested for X-ray diffraction at synchrotron facilities PETRA III, Germany; MAXIV, Sweden; ESRF, France; and SLS, Switzerland. Room-temperature screening on AQP10 was carried out at SLS. For cryogenic data collection, the crystals were cryoprotected with glycerol, while the ambient temperature tests were done in capillaries mounted at the beamline. The diffraction limit was assessed visually by inspection of the diffraction patterns.

## Results and Discussion

### Growing Larger Crystals Applying Available X-ray Crystallization Conditions

NMX requires large and well-diffracting crystals, ideally diffracting beyond 2.5 Å.^10^ This is challenging for most membrane proteins due to typically limited yields of proteins, the presence of detergents that interfere with target stability and crystal packing, and a large solvent fraction in the crystals. Previously, we have generated X-ray structures of AQP7 and AQP10 (Figure 1A,B) using crystals diffracting to 1.9 Å (Figure 1C) and 2.3 Å (Figure 1D), respectively. Both proteins were crystallized in glucoside detergents.^8,9^ Here, we aim to optimize these conditions to retrieve large crystals suitable for NMX.

### Crystallization Setup and Time

**Figure 2:**
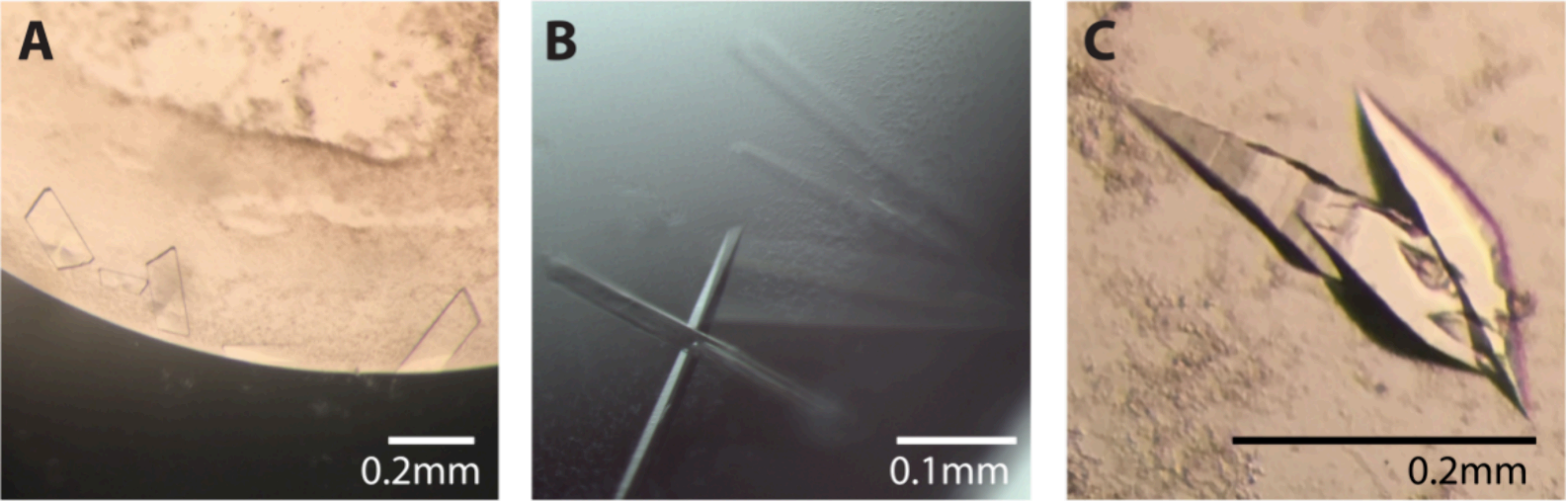
Larger crystals of AQPs by method optimization. (A) AQP7 crystals grown by sitting drop vapor diffusion, reaching up to 0.15 mm × 0.1 mm thin plates with a bigger drop volume. (B) AQP10 crystals grow larger when drops are left for six months. (C) Macroseeding of small AQP7 crystals into a fresh, unequilibrated hanging drop, which grew up to 0.3 mm in length.

The crystals of AQP7 and AQP10 diffract well,^8,9^ making these target proteins promising candidates for neutron diffraction studies. The crystallization conditions for AQP7 and AQP10 used for X-ray data collection were applied as a basis for the crystallization method optimization. Crystallization for NMX often requires the sitting drop vapor diffusion method to accommodate the increased drop sizes typically required for the generation of larger crystals. In light of this, the crystallization conditions were transferred from the hanging drop to sitting drop format, thereby affecting the surface tension of the drop and the equilibration rates. In this format, AQP7 crystals nucleated and increased in size over several weeks of incubation in drops twice as big compared to the hanging drop setup. However, the majority of the crystals grew primarily in two dimensions, resulting in thin plates with increased surface area (approximately 0.15 × 0.1 mm, Figure 2A). Another approach is to allow crystals to grow for longer periods of time. This was applied for AQP10 using the original conditions; however, instead of extracting the crystals after 2 days of incubation, drops were left for up to six months. Interestingly, the crystals changed from needles to 2 mm long rods, with a clear three-dimensional form (Figure 2B, Table 1).

**Table 1:** Crystallization Conditions/Optimization Strategies and Diffraction Limit at Cryogenic Temperatures for AQP7-ΔC, AQP7-ΔNΔC, and AQP10-ΔNΔC

variant/detergent	crystallization condition	protein conc. (mg·mL^–1^)	temp (°C)	final crystal size^a^ (mm)	final diffraction	modification
AQP7-ΔNΔC/OGNG	37% PEG 200	9	4	0.15 × 0.1	n/d	sitting drop crystallization macroseeding
	0.085 M Tris pH 8.5			0.3	n/d	
	0.8% OG					
AQP7-ΔC/DM	25% PEG 400	8.9	4	0.1	14 Å	additive screening 18 °C 7 mg·mL^–1^
	0.1 M HEPES pH 7					
	0.1 M NaCl					
	0.1 M Li_2_SO_4_					
AQP7-ΔC/DDM	30% PEG 400	7.3	18	0.1 × 0.05	7 Å	5.3 mg·mL^–1^ additive screening 30 °C
	0.1 M Glycine pH 9.3			0.2	weak diff.	
	0.1 M Li_2_SO_4_			1	weak diff.	
AQP7-ΔC/OM	30% PEG 400	3.6	18	0.05	8 Å	n/a
	0.1 M Glycine pH 9.3					
	0.1 M Li_2_SO_4_					
AQP7-ΔNΔC/NG	53% v/v PEG 400	4	18	0.03	weak diff.	additive screening
	0.1 M HEPES pH 7.5					
	0.2 M CaCl_2_ dihydrate					
AQP10-ΔNΔC/NG	17–22% PEG2KMME	2.5	18	0.01 × 0.01 × 2	1.8 Å	removal of additive (NTG)
	0.01 M MES-NaOH pH 6					longer incubation (6 months)
	2.5–10% glycerol					
AQP10-ΔNΔC/NTG	18–19% PEG550MME	8	18	0.75 × 0.75	10 Å	longer incubation (weeks)
	0.1 M HEPES pH 7					
	0.2 M NaCl					
	5% glycerol					

a Longest dimension(s) reported. Most crystals were not able to be measured in the third dimension, while they were stuck to the coverslips.

### Seeding

During optimization of the AQP7 crystallization conditions, we observed that crystals continued to grow in drops for several weeks after the initial harvesting, and unexpectedly, the new crystals were of better quality compared to the initial ones. The fact that new crystals appeared suggests that crystal formation may have benefited from unintentional seeding. In this light, single crystals were seeded into fresh, unequilibrated drops, resulting in additional growth of the crystal in all dimensions, up to approximately 0.3 mm in length (Figure 2C, Table 1), representing an interesting approach for reaching crystals with sizes suitable for NMX.

### Growing Larger Crystals Using New Optimized Conditions

To grow well-diffracting crystals of integral membrane proteins, it is crucial to select an optimal detergent to keep the protein in solution, which, at the same time, enables crystal formation. Glucosides and maltosides are examples of nonionic detergent types that are considered mild, which have yielded protein crystals of AQPs and other membrane proteins in the past.^14^ Generally, surfactants with short alkyl chains have a worse ability to stabilize proteins extracted from a membrane, whereas long chains may interfere with crystal packing. Thus, it is relevant to investigate the solubility and stability of different protein variants in varying detergents when aiming for larger crystals.

### Solubility and Stability of Human Aquaglyceroporin Variants

**Figure 3:**
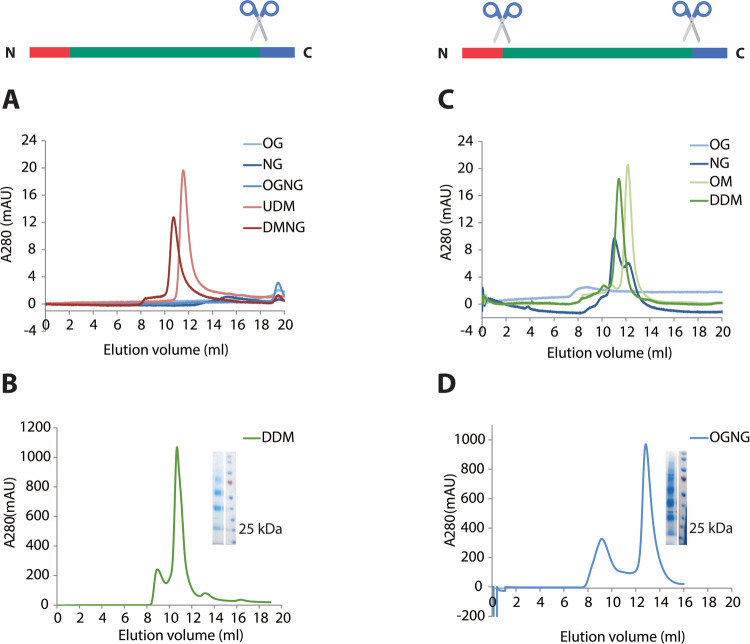
SEC profiles for AQP7-ΔC and AQP7-ΔNΔC in various detergents. (A, B) SEC profiles for AQP7-ΔC. (A) Small-scale runs and (B) large-scale purification in DDM with corresponding SDS-PAGE as an insert. The AQP7-ΔC monomer band migrates to around 25 kDa, and higher oligomeric states are also present. (C, D) SEC profiles for AQP7-ΔNΔC. (C) Small-scale runs and (D) large-scale purification in OGNG with corresponding SDS-PAGE as an insert. AQP7-ΔNΔC monomer band migrates to around 25 kDa, and higher oligomeric states are also present. Schematic sequence of wtAQP7 shown above the chromatograms with scissors indicating cleavage sites for reference.

Several detergents were evaluated by applying SEC on variants of AQPs (wild-type AQP7 (wtAQP7), C-terminally truncated (AQP7-ΔC), and N- and C-terminally truncated (AQP7-ΔNΔC)) and AQP10 (wild-type AQP10 (wtAQP10) and N- and C-terminally truncated (AQP10-ΔNΔC)) to keep the proteins stable and homogeneous in solution. Most of the AQP structures deposited in PDB are obtained with glucoside detergents.^15−19^ Likewise, the published X-ray structures of AQP7 and AQP10 were generated using purification and crystallization of AQP7-ΔNΔC in OGNG,^8^ as well as of AQP10-ΔNΔC in NG with NTG as an additive.^9^ To investigate how other detergents affect the crystallization, a C-terminally truncated variant, AQP7-ΔC, was purified in several glucoside and maltoside detergents (OG, NG, OGNG, UDM, DMNG, and DDM). Interestingly, none of the glucoside detergents were suitable for this construct, not even OGNG, which was used for the AQP7 structure derived from AQP7-ΔNΔC (Figure 3A,B). However, in addition to the previously analyzed OGNG (Figure 3D), the maltosides DDM and OM (the maltoside equivalent of OG), as well as, to some extent, the glucoside NG (but not OG) sustained a complementary double-truncated variant, AQP7-ΔNΔC, in solution (Figure 3C).

**Figure 4:**
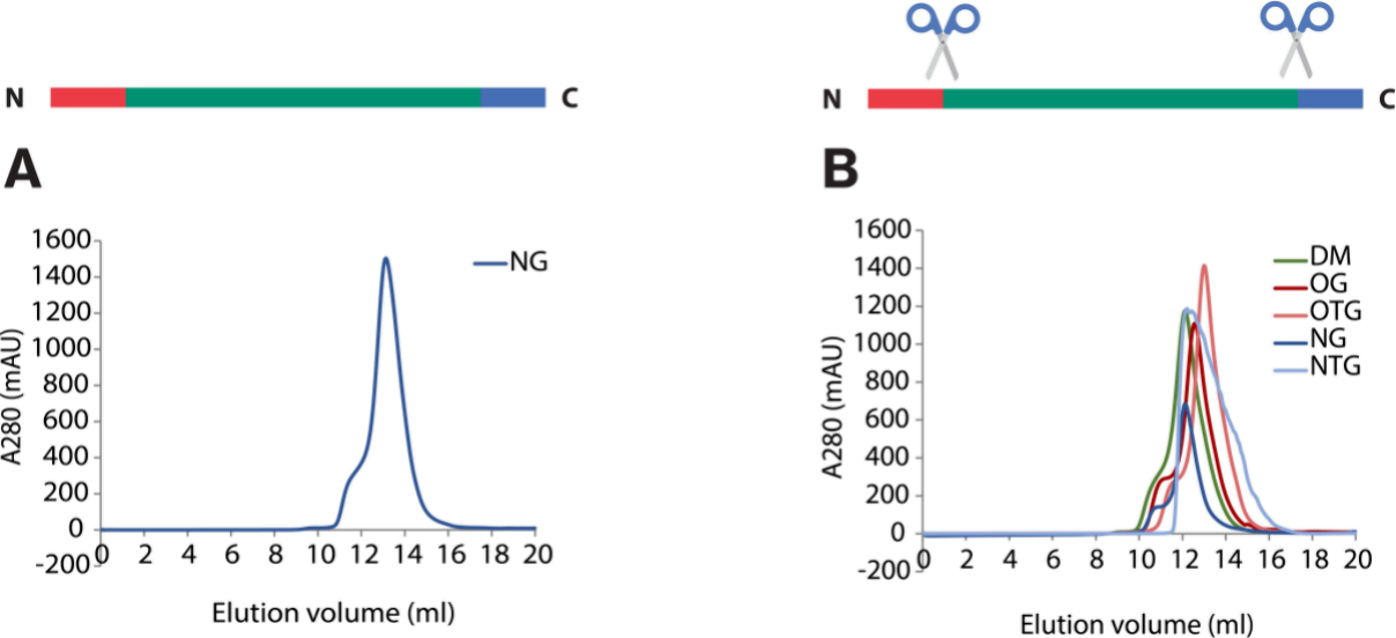
SEC profiles for wtAQP10 and AQP10-ΔNΔC in various detergents. (A) SEC profile for wtAQP10 in NG. (B) Large-scale purification profiles for AQP10-ΔNΔC in DM, OG, OTG, NG, and NTG. Schematic sequence of wtAQP10 shown above the chromatograms with scissors indicating cleavage sites for reference.

In contrast to AQP7, wtAQP10 and AQP10-ΔNΔC provided the required amounts and performed well in a variety of detergents (both glucosides and maltosides) (Figure 4). Upon successful isolation, affinity purification was performed in the presence of DM and exchanged using SEC with different glucoside detergents (OG, NTG, OTG), known to facilitate AQP crystallization. OG and OTG, as well as the previously analyzed NG detergent, yielded monodisperse profiles (Figure 4A,B). Conversely, NTG produced rather broad SEC profiles, perhaps due to the poor stability of the detergent in solution. Collectively, this demonstrates that AQP10 constructs are tolerant to a relatively wide range of detergents and that detergent tails with eight carbons are sufficient for our double-truncated AQP7 and AQP10 constructs, while longer tails are necessary for the single truncation or the wild-type proteins.

### Crystallization of the AQP7-ΔC and of AQP7-ΔNΔC Variants under New Conditions

**Figure 5:**
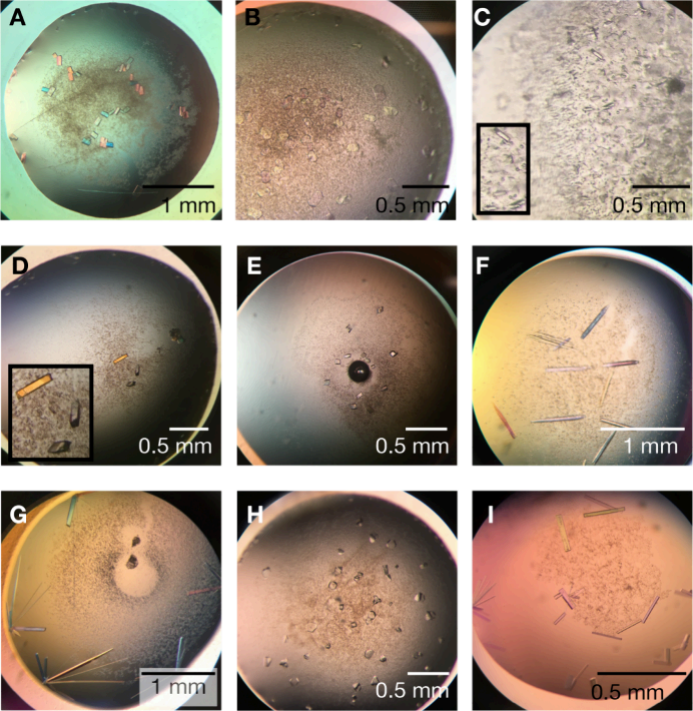
Crystals with different truncations and detergents of AQP7. Crystals of (A–G) AQP7-ΔC and (H, I) AQP7-ΔNΔ*C* grown by hanging drop vapor diffusion. (A) Example of crystals in DM, longest dimension ∼ 0.1 mm. (B, C) Initial hits for DDM diffracting to 16 Å. (D–G) Optimization of the crystallization conditions in B and C. (D) Tripao as an additive, longest dimension ∼ 0.2 mm. (E) Nonylthiomaltoside as an additive. (F, G) Crystals without additive grown at 30 °C, longest dimension ∼1 mm. (H, I) Crystals grown under optimized OGNG conditions, resulting in smaller crystals with different morphology compared to the initial hit. (I) Crystal grown in NG, up to 0.3 mm in the longest dimension.

Based on the results from the detergent analyses, AQP7-ΔC was assessed for its ability to yield crystals for neutron studies. AQP7-ΔC was successfully crystallized in several detergents (OM, DM, UDM, DDM, DMNG), precipitants, pH, and the presence of various additives (Figure 5, Table 1). Initial hits in DM and DDM (Figure 5A–C) diffracted to ∼16 Å and were then further optimized. Crystals in DM could be improved marginally in resolution when crystallized at 18 °C instead of 4 °C (∼14 Å) and in the presence of the secondary (additive) detergent Cyclofos TM-3 (Table 1). Similarly, AQP7-ΔC was prone to crystallization in DDM; however, the diffraction quality remained poor after extensive optimization efforts. However, the presence of additives in the drop changed crystal morphology, and the crystals in the presence of DDM grew to approximately 0.2 mm in the longest dimension (Figure 5D,E). In addition, larger crystals, up to 1 mm long, grew at 30 °C, appearing after 2–3 weeks (Figure 5F,G). Optimization of the crystallization conditions did not yield better diffracting crystals, although the crystal morphology changed (Figure 5H). On the other hand, crystallization in NG resulted in larger crystals, up to 0.3 mm; however, the crystals were less well-packed (Figure 5I, Table 1). This suggests that shorter alkyl chains promote better crystal packing, while longer alkyl chains improve the crystal size, as also seen for the AQP7-ΔC variant. This could be explained by the fact that shorter alkyl chains favor an increased number of protein–protein contacts, which, in turn, results in better crystal packing, while longer alkyl chains result in more stable proteins in solution but less protein/protein contacts. For the aquaporins studied here, this appears to be linked to the formation of larger crystals. We speculate that increased crystal size with longer alkyl chain detergents may relate to (1) more stable protein in the crystallization drop and (2) lower nucleation rate, two factors that both can contribute to crystal growth (particularly in cases with longer crystallization periods) as more protein is available.

### Crystallization of the AQP10-ΔNΔC Variant under New Conditions

**Figure 6:**
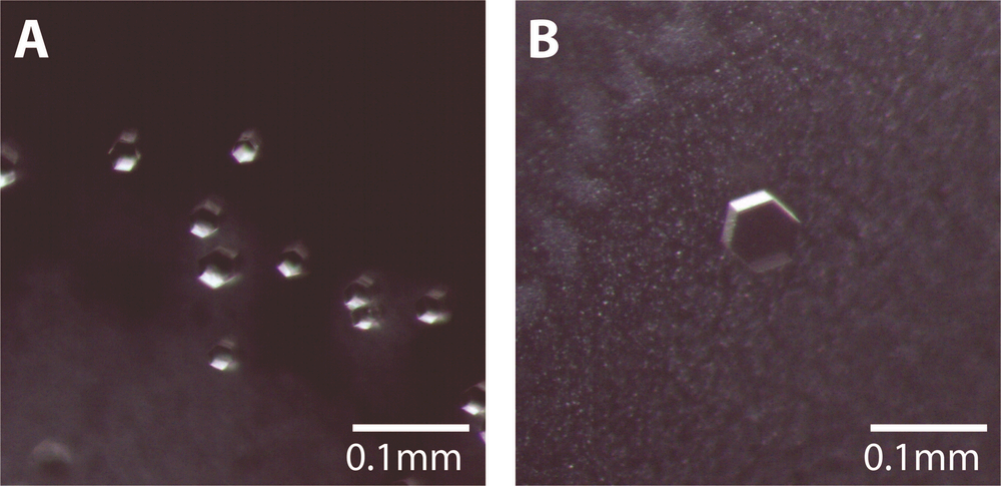
Crystals of AQP10-ΔNΔC purified in NTG grown by hanging drop vapor diffusion. (A) Crystals grown in NTG conditions resulted in crystals with different morphology and smaller in size than when grown in NG. (B) Crystals grown in optimized NTG condition resulted in larger crystals compared to initial hits.

Equivalent to AQP7, AQP10 variants were approached (wtAQP10 and AQP10-ΔNΔC) to analyze new crystallization conditions than those employed for determining the X-ray structure^9^. We tested the detergents OG, OTG, and NTG, all demonstrating different degrees of stabilizing effect on the protein (Figure 4B). The initial hits obtained in OG and OTG as the main detergent did not result in diffraction beyond 15 Å and only in needle-shaped crystals. Therefore, we discontinued work with those surfactants. In the original crystallization condition, NTG was used as an additive. When omitting NTG, the crystals occasionally grew larger, which coincided with improved resolution of the crystals, diffracting to 1.8 Å (Figure 2B, Table 1). However, these crystals were not easily reproduced and therefore not optimal for further optimization. Furthermore, these crystals demonstrated increased size in one dimension, while NMX studies benefit from larger more square-like shapes. Moreover, we attempted to lower the temperature for all crystallization conditions to 4 °C; however, those crystals only diffracted to 10 Å or worse and were rather small compared to the hits obtained at 18 °C. Interestingly, despite the broad SEC profile, hits obtained for NTG-purified AQP10-ΔNΔC resulted in three-dimensional hexagonal crystals diffracting initially to 10 Å (Figure 6A, Table 1). Applying the same strategies as those exploited for NG-purified AQP10-ΔNΔC (specifically the longer incubation time mentioned above), we were able to increase the size of the crystals, although with no increase in diffraction capacity (Figure 6B).

## Conclusions

Neutron diffraction crystal structures enable the determination of hydrogen atom positions in proteins, which are typically absent in structures determined by using X-ray crystallography. Water and glycerol molecules line the pores of AQP7 and AQP10; however, to fully understand the substrate conducting and proton exclusion mechanisms, locating hydrogen atoms and hydrogen bonds is necessary. We have previously obtained crystals of AQP7-ΔNΔC and AQP10-ΔNΔC diffracting to 1.9 and 2.3 Å and subjected these crystal forms for optimization efforts for obtaining larger crystals suitable for neutron diffraction data collection. Two strategies were executed, first amending the previously obtained X-ray conditions to analyze whether larger crystals can be formed and, second, production of new variants of the AQPs to test if other detergents are compatible with solubility and increased crystal size.

Applying the X-ray conditions, we conclude that the best strategies to retrieve large crystals are long incubation times, large drops, and seeding. However, to obtain different crystal packings or large crystals (millimeter size), new constructs and new detergents were required. For AQP7, large crystals were grown in the presence of maltoside detergents, up to 1 mm in one of the dimensions. Interestingly, these crystals were formed upon increasing the temperature to 30 °C. Attempting crystallization of AQP10-ΔNΔC in different detergents did not yield any diffraction improvements over the original detergent NG. However, crystallization in the presence of NTG (replacing NG as the main detergent) provided a promising morphology change with a hexagonal crystal. Although the chemical structures of NG and NTG are very similar, they vary slightly in their chemistry as NTG has a thioether group linking its polar head with the hydrophobic tail (ether in NG). This means that NTG is slightly less polar than NG. Nonetheless, it is difficult to fully resolve the molecular mechanism explaining the differences in the crystal morphology observed by NTG. Clearly, NTG in a concentration-dependent manner influenced crystal packing, forcing the AQP10 molecules to pack differently. As aquaporins have only limited portions exposed to the soluble environment (tails and loops), AQP crystals may be relatively susceptible to such detergent-induced changes in crystal packing. These hexagonal crystals were applied for 18 °C data collection; however, the initial attempts suggested that they are not stable outside cryogenic conditions. Typically, larger crystals should give a stronger signal; however, here, the larger crystals, for both AQP7 and AQP10, resulted in weaker diffraction. This was even the case when crystals grew under very similar conditions (Table 1). This would suggest that, here, larger crystals are more disordered than smaller crystals or more susceptible to mechanical stresses in mounting; however, as the diffraction for the largest crystals was very weak, it is not possible to confirm this by inspecting the spots in the diffraction patterns. This work lays the foundation for continued neutron crystallization efforts targeting AQPs and other membrane proteins. The neutron structure of an aquaglyceroporin is a crucial complement to further our understanding of its selectivity and conducting mechanism.

## Data Availability

The authors confirm that the data supporting the findings of this study are available within the article.
